# The Latest Treatment Interventions Improving Mental Health Outcomes for Women, Following Gender-Based Violence in Low-and-Middle-Income Countries: A Mini Review

**DOI:** 10.3389/fgwh.2021.792399

**Published:** 2021-12-16

**Authors:** Lily St. John, Rebecca Walmsley

**Affiliations:** ^1^School of Medical Sciences, University of Manchester, Manchester, United Kingdom; ^2^School of Medicine, University of St Andrews, Fife, United Kingdom

**Keywords:** gender-based violence, mental health, treatment, interventions, low-and-middle-income countries

## Abstract

Gender-based violence (GBV), specifically violence against women, is a worldwide pandemic. Prevalence is further escalated in low-and-middle-income countries and in humanitarian crises. Survivors are left with a combination of post-traumatic stress disorder, depression and anxiety. These mental health disorders lead to further morbidity and mortality. Despite its high prevalence and co-morbidities, gender disparities and mental health stigma globally lead to few interventions developed for this population. The aim of this review is to highlight the mental health interventions developed in the past 5 years, for women following GBV in low-and-middle-income countries. It aims to discuss their efficacy and controversies when implemented into healthcare systems, understand the gaps that remain in the field and suggest future research developments. A thorough literature search revealed 16 new interventions available for improving mental health outcomes for women following GBV in low-and-middle-income countries. Following an in-depth evaluation of the papers, one intervention was successful in effectively implementing treatment into healthcare systems—“PM+.” However, it proved only to be effective in the short term. Further research must be done for improving long-term mental health outcomes. Results demonstrated poor follow-up for women engaging in group therapy. The review also highlights community workers were used in service delivery to reduce barriers accessing care. No interventions proved effective in humanitarian crises, despite GBV escalated in these settings. There are very few interventions available in comparison to the prevalence of this global health issue. Therefore, this review encourages further research and improvements in mental healthcare interventions following GBV.

## Introduction

Gender-based violence (GBV), particularly violence against women, is a global pandemic. Violence against women (VAW) between the ages of 15-44, causes more morbidity and mortality than malaria, traffic accidents and cancer combined (1). Estimates suggest that around one in three women will experience sexual violence in their lifetime (2). VAW occurs in every country, community and culture, regardless of race, status or wealth; however, it is more prevalent in low-and-middle-income countries due to further inequities (3). A report revealed statistics of VAW as high as 60% in the Democratic Republic of Congo and 65% in South Sudan (4)—twice as much as the global average. Another survey conducted in Bangladesh reports 61% of men strongly agreed with the statement, “there are times when a woman deserves to be beaten” (5). These gender disparities also limit access to healthcare for women. A study in Somalia estimates only 10% of female rape victims seek medical attention (6). Effective healthcare services following GBV also remains rare, especially mental health facilities.

The lasting effects of the mental health disorders and the predisposed nature of these vulnerabilities to another attack means the psychiatric care for patients is critically important (7). Mental health disorders which survivors suffer from (post-traumatic stress disorder (PTSD), anxiety and depression) hugely affect their quality-of-life as well as affecting the health outcomes of their children (8). Additionally, a study looking at the effects of sexual violence and mental health on wound healing and inflammation, found that there were immune changes in the female reproductive tract in those patients with chronic sexual abuse and depression (9). Another report further emphasizes its impending implementation describing, without mental-health treatment in the long-term patients will experience psychotic episodes, anxiety and depression and may attempt suicide (10).

Limited women's rights globally (11), combined with global stigma of mental health disorders (12) has led to a critical gap in the literature on this topic. Many scholars have recently noted the need for intervention in this field (13) yet have not reviewed and identified the best approach forward. This review will collate the most recent studies aiming to improve the mental health outcomes for this population and will answer what are the new most effective methods in delivering this care. This will fill a gap in the literature, encourage development of research and interventions, and strengthen the support for survivors living with mental health disorders in low-and-middle-income countries.

## Methods

On establishing the gap of interventions aiming to improve mental health outcomes for women following GBV in low-and-middle-income countries, a literature search was conducted using four databases: *PubMed, Ovid Medline, PsychInfo*, and *Global Health*.

The following key words were used: gender-based violence^*^—*violence against women, sexual violence*; low-and-middle-income countries; mental health^*^—*psychological, psychosocial;* treatments^*^—*interventions*.

The definition of GBV is, “*any harmful act directed against individuals or groups of individuals on the basis of their gender. It may include sexual violence, domestic violence, trafficking, forced/early marriage and harmful traditional practices.” (United Nations Human Rights)* (14). This paper specifically focuses on GBV against women. Table 1 outlines the inclusion and exclusion criteria.

**Table 1:** Inclusion and exclusion criteria.

	**Inclusion criteria**	**Exclusion criteria**
Population	- Women affected by gender-based violence in low-and-middle-income countries - Low-and-middle-income countries with humanitarian crises at the time of study (both natural disasters and conflict settings) - Civilians - Women over 16 years of age	- Any other gender - High income countries - Military personnel - Psychological trauma not associated with gender-based violence
Interventions	- Interventions established specifically for women following gender-based violence	- Screening interventions rather than treatment interventions
Outcomes	- Interventions aiming to improve any mental health outcome - Qualitive and quantitative measured outcomes	- Interventions aiming to improve only physical health outcomes
Study design	- Peer reviewed studies (including RCTs, cohort studies, case-control studies) - Studies from 2015-2020 - All languages	- Grey literature and non-peer reviewed studies - Studies published before 2015 - Studies not completed or study protocols

Database search filters included: 2015-2020; all languages; female; and peer-reviewed literature. The five-year period was chosen to obtain only the most recent research.

**Figure 1:**
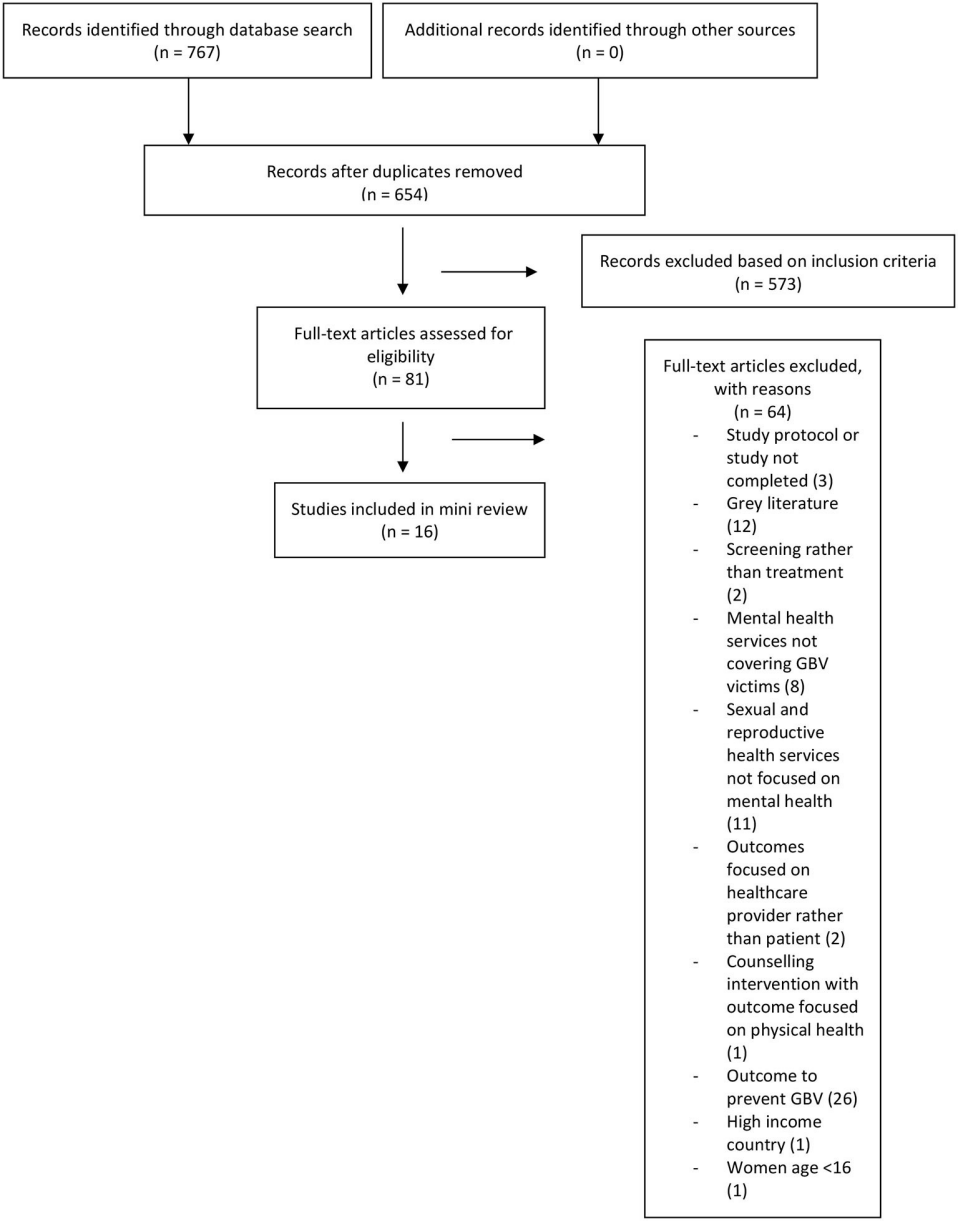
Search strategy (15).

Seven hundred and sixty seven papers in total were extracted to Endnote library and screened using their titles and abstracts. 81 papers were screened using the full article. Figure 1 outlines the search strategy.

## Results

Sixteen papers were identified with interventions aiming to improve mental health outcomes for women following gender-based violence in low-and-middle-income countries. Five were Randomized Controlled Trials (RCT), two pilot RCTs, two cross-sectional studies, six cohort studies and one case study. There was a large variation in participant numbers per trial, ranging from one (in the case study) to 14,730 (in a cross-sectional study).

The screening process demonstrates there are limited interventions currently available to improve mental health outcomes for women following GBV in low-and-middle-income countries and highlights that further interventions should be formed. However, the following reviews the 16 new treatments that are available. It discusses their respective effectivity, strengths and limitations, compares their results as well as highlights areas for future development.

## Discussion

### Interventions Ineffective in Improving Mental Health Outcomes

Five papers revealed ineffective results in improving mental health outcomes for women following gender-based violence, three of these trialed in conflict settings. GBV is more prevalent when conflict and crises occurs in low-and-middle-income countries (16). One study explored whether narrative story telling in safe houses in Afghanistan could improve mental health outcomes for women following GBV (17). This intervention recruited the cohort externally, minimizing bias; it also explored all relevant mental health outcomes. However, due to political issues surrounding the safe houses at the time, no data was collected leading to inconclusive results. Short-term follow up and a small population size (*n* = 20) also limits this study.

An intervention in Côte D'Ivoire tested a “gender norms group discussion” to reduce PTSD following intimate partner violence (IPV) compared to an economic intervention (18). Overall, no significant difference was seen in intervention compared to control. This study was also interrupted by conflict with the treatment arm postponed a for a year. Patient and providers were not blind to treatment, with participants given their allocations through a broadcast in the community. This created a potential confounding bias. These significant limitations prevent the implementation of this treatment plan globally; however, the study did maintain 95.9% of participants through to the end of the trial and upheld similar demographics in each group (e.g., religion, ethnicity, marital status, and age). Group Cognitive Processing Therapy (CPT) was tested during conflict in the Democratic Republic of Congo in comparison to individual treatment, to assess whether psychotherapy can have an impact on perceived stigma and resultant mental health disorders (19). Findings showed Group CPT can reduce feelings of stigma only in the short term compared to the control arm (*p* = 0.02). However, statistical significance was not maintained at 6 months. Results also demonstrated reduced stigma had no effect in improving mental health outcomes. Furthermore, not all patients were accounted for at the end of the trial, with 33 and 23% missing the assessment at first and second follow up, respectively. Despite not improving mental health outcomes, the study did address a clearly focused issue—women who have experienced GBV who have resultant mental health disorders and some impairment in functioning—reliable score scales were used to assess these.

The failed delivery of the three interventions above means our understanding and ability to implement interventions in conflict settings still remains limited. This review therefore calls for further research to implement the appropriate interventions needed in crises.

Another intervention in Dadaab Refugee Camp, Kenya (20) concludes that significant challenges faced by refugee community workers impaired provision of effective care. This finding is supported by the literature (21). In light of this, community members may not provide the most effective mental health treatment, despite frequent trials in interventions. The participants of this study were recruited purposively, to provide a range of ages, but the small sample size (*n* = 20) was not justified. A study in Mombasa, Kenya, also tested a community worker care model for survivors of sexual violence (22). Differing from the previous interventions, it was delivered in a hospital setting. On implementation of the trial, risk factors were addressed; ensuring patients were safe due to perpetrators often inhabiting in close proximity to victims. This is important due to frequent cases of close perpetrator to victim proximity in low-and-middle-income countries (23). Furthermore, the sample size was large (*n* = 3,973). The sample was also representative of the population in question, including a range of ages and encompassing all necessary psychological outcomes. However, poor follow up (19% returned for the second session and 1.54% attended all five sessions) led to inconclusive results in determining effectivity of treatment. It is difficult to assess if the barriers in accessing this care were community workers or the hospital setting. A trial should be conducted to compare settings to determine the most accessible for this population.

### Successful Community Delivered Care

Several successful interventions followed a similar principal: providing community delivered care in attempt to overcome barriers that prevent patients from accessing treatment.

A combined Cognitive Processing Therapy (CPT) and Advocacy Counseling intervention provided by lay refugee staff in Tanzania (24) was trialed to decrease psychological stress in women following IPV. On average, two-thirds of these sessions were attended, and results concluded that although there were many challenges faced, it was a feasible intervention to improve the mental health of IPV survivors. CPT has been used before for sexual assault victims (25), but the literature states it is not appropriate for IPV victims and should be used only in acute trauma (26). This combined intervention may be a successful approach in treating IPV survivors. However, the cohort was small (n=60) and was only recruited from one camp “zone.” Community facilitators were also not blind to the intervention, revealing a potential bias. Finally, the interviews were taken directly following the 8-session intervention, meaning no long-term follow up was obtained. Although an encouraging paper revealing successful results, the limitations prevent the application of this intervention to larger scale.

Another successful community delivered care model was the Problem Management Plus (PM+) five-session behavioral intervention in Kenya (27). Three months from baseline assessment the results demonstrated an improvement in psychological disorders compared to Enhanced Usual Care (EUC) (*p* = 0.001) for women following GBV. Minimizing bias, the assignment of patients to EUC or “PM+” was randomized and conducted by staff independent to the trial. The successes of this randomized controlled trial are evident from being an intervention recommended by the World Health Organization (28). Research should be conducted to improve the length of treatment effects over 3 months. Importantly, women in this study were identified for psychological distress and disorders prior to treatment. Future interventions should follow the same model and assess GBV victims for all clinically relevant mental health outcomes before initiating management.

An evaluation of a rural response system in effectively treating depression was conducted in Ghana using Community Based Action teams (29). The intervention, part of “*What Works?”* (30) preventing violence scheme, showed a significant reduction in depressive symptoms following intervention compared to no action (*p* < 0.01). The study encompassed a large population size, studied over four districts in Ghana. It also spanned over 24 months, contrasting to the shorter 3-month studies seen above. Although helpful for treating depressive disorder in GBV, it does not address PTSD or anxiety, despite PTSD occurring most frequently in these patients, and anxiety often occurring (31).

The Ushindi Model was a multidisciplinary community faith-based service in the Democratic Republic of Congo (32) which aimed to improve mental and physical health outcomes for women following GBV. Results show this project has continued beyond the given time frame of the trial, with 71.5% of participants attending psychosocial services provided. The intervention spread over 10 “health zones,” covering over one million people. These areas were selected based on limited GBV care facilities. Similar to other interventions, this trial was delivered by community workers. This perhaps owes to its sustainability. However, this is difficult to conclude given the challenges faced by other community delivered care. The key limitation is the omission of the local community members survey, with qualitative data generated from providers of the care. Conclusions cannot therefore be definitively made as to whether patients feel this intervention successfully improves mental outcomes following GBV.

### Other Mechanisms to Overcome Barriers in Accessing Care

The Healthy Activation Program (HAP) in Goa (33) focused on the decrease in depressive symptoms in GBV victims following a culturally adapted behavioral treatment, in comparison to Enhanced Usual Care (EUC). The literature highlights the need for female mental health interventions to be adapted depending on location, religion and beliefs (34). Results of HAP showed a reduction in depression at 12 months, however there was no difference between both groups. Allocating patients randomly and treating groups equally created minimal bias. This paper is again encouraging for treating depressive disorder, but all clinically important outcomes were not measured, including PTSD and anxiety.

Another trial which focused on overcoming barriers to accessing psychosocial care was delivered using a mobile service delivery intervention to refugees in Lebanon, provided by *International Rescue Committee* (IRC) (35). The mobile service delivery is something the IRC has provided before in low resource settings (36), achieving successful results. Participants reported reduced negative feelings, and reduced feelings of isolation or stress. However, results do not indicate the number of patients reporting this, limiting its reliability. Further limitations to this study include a small cohort (*n* = 56) recruited by IRC staff, creating potential bias.

### Interventions Delivered by Medical Professionals

The “Family Support Medical and Counselling Centre” in Papua New Guinea, facilitated by *Medicines Sans Frontiers* (37) provided psychological first aid and informative counseling. The observational study demonstrated improvement in patient's psychosocial presenting complaints following two or more counseling sessions (*p* = 0.001). The cohort of patients was large (5,9892) and data from the Center was analyzed over a 3-year period (2010-2013). Furthermore, the majority of patients were walk-ins, highlighting there was no bias in selecting the cohort for this study. However, the outcomes measured did not directly assess all key mental health disorders. There was also poor follow up with only 6% attending more than three counseling sessions. Research shows that better mental health outcomes are achieved with higher frequency of treatment sessions (38).

The nurse delivered intervention, in South America (39) proved statistically significant treatment effects in improving mental quality of life for IPV survivors 3 months post intervention but failed to at 15 months (*p* = 0.3). Similarly, improving the frequency of sessions between the 3- and 15-month gap may improve the longevity of the treatment effect. This study recruited a large population size (*n* = 950). Furthermore, the trial randomly allocated participants to respective groups. However, neither nurses nor patients were blind to treatment groups. Another cofounding bias was providing monetary incentives. This may also have improved the mental health for both groups irrespective of treatment.

Finally, three studies explored the intervention “ImpACT” (Improving AIDs Care After Trauma), a counseling approach to improve mental health outcomes following GBV and study its effect on HIV treatment uptake. The pilot RCT implementing “ImpACT” (40) showed reduction in PTSD symptoms and an increase in anti-retroviral therapy uptake 3 months from baseline compared to the control arm. However, at 6 months the decrease in PTSD symptoms was not significantly different to the control and there was poor uptake of anti-retroviral therapy; another intervention where treatment effects are beneficial in the short term but do not improve long term mental health outcomes. Participants were randomly allocated to either group, and allocation was blind to staff involved. Limitations to “ImpACT” include only measuring changes to PTSD, and not accounting for all patients when measuring results. Furthermore, monetary incentives were also used for this study creating potential cofounding bias. Despite this, the case study of “ImpACT” (41) showed the patient's PTSD symptoms dropped from 66/80 to 18/80 and depression scores from 52/60 to 4/60 at 6 months. Though one case report does not deter from the results of the pilot RCT. Another study to explore mechanisms of change in mental health outcomes and barriers to counseling revealed “positive thinking” following the “ImpACT” intervention (42), however it also showed that only 25.8% of women attended the group sessions—demonstrating a further study with restrictions to group therapy for women.

## Conclusion

This review highlights certain factors that are vital for deciding and implementing interventions in low-and-middle-income countries. One factor is appropriate assessment of all relevant psychiatric disorders prior to commencing treatment. The PM+ intervention (26) assessed patient needs prior to treatment, perhaps owing to the success of the treatment. Two further factors which are vital for implementing interventions are: frequency of care provision, and appropriate setting. Supported by the literature, it demonstrated a higher frequency of treatment sessions delivers better mental health outcomes. For an appropriate setting, it is clear one-to-one therapy is more successful for adult women compared to group therapy. This review also highlights the need for further research in other aspects of appropriate setting. Different locations must be compared, whilst maintaining continuity in other variables to determine the most appropriate setting to deliver this care. These may include: safe houses; hospital; mobile service delivery; disguised locations; and obvious clinics.

Service delivery personnel is another factor that remains unclear following review of these interventions. Some interventions argue community workers reduce barriers in accessing care, while some highlight the challenges this causes, and resultant poor patient follow up.

However, when deciding both delivery personnel and setting, there must be minimal patient barriers in accessing care.

This review highlights the need for future research and encourages ongoing intervention development for this population. Based on the review, we lay down the following four principles to use when developing future interventions to improve mental health outcomes for women following GBV: (I) Accessibility—barriers in accessing care must be removed where feasibly possibly; (II) Effectivity—the intervention must be effective in improving mental health outcomes; (III) Longevity—the intervention must aim to improve long-term mental health outcomes; (IV) Equality—each patient must be treated equally and with respect.

Finally, this review encourages the development of prevention and awareness programs to help reduce GBV prevalence, as well as educate communities on available care.

## Author Contributions

LSJ and RW conceived the idea for the review, contributed to the article, and approved the final version. LSJ wrote the initial draft. RW revised the review. Both LSJ and RW edited the review. All authors contributed to the article and approved the submitted version.

## Conflict of Interest

The authors declare that the research was conducted in the absence of any commercial or financial relationships that could be construed as a potential conflict of interest.

## Publisher's Note

All claims expressed in this article are solely those of the authors and do not necessarily represent those of their affiliated organizations, or those of the publisher, the editors and the reviewers. Any product that may be evaluated in this article, or claim that may be made by its manufacturer, is not guaranteed or endorsed by the publisher.

## Abbreviations

GBV: gender-based violence
VAW: violence against women
IPV: intimate partner violence
HIV: human immunodeficiency virus
RCT: Randomized controlled trial
IRC: International Rescue Committee.
